# Human Tumor Antigens and Cancer Immunotherapy

**DOI:** 10.1155/2015/948501

**Published:** 2015-06-16

**Authors:** Nathalie Vigneron

**Affiliations:** ^1^Ludwig Institute for Cancer Research, 1200 Brussels, Belgium; ^2^WELBIO (Walloon Excellence in Life Sciences and Biotechnology), 1200 Brussels, Belgium; ^3^de Duve Institute, Université Catholique de Louvain, 1200 Brussels, Belgium

## Abstract

With the recent developments of adoptive T cell therapies and the use of new monoclonal antibodies against the immune checkpoints, immunotherapy is at a turning point. Key players for the success of these therapies are the cytolytic T lymphocytes, which are a subset of T cells able to recognize and kill tumor cells. Here, I review the nature of the antigenic peptides recognized by these T cells and the processes involved in their presentation. I discuss the importance of understanding how each antigenic peptide is processed in the context of immunotherapy and vaccine delivery.

## 1. Introduction

Active cancer immunotherapy aims at activating the adaptive immune system of cancer patients to destroy tumors and prevent their recurrence. Key effector cells are the antitumor cytolytic T lymphocytes (CTL), which are poised to recognize and kill tumor cells. Strategies designed to activate antitumor CTL found in the blood and in the tumors of cancer patients are therefore promising for the development of strong and long-lasting antitumor responses. In the recent years, cancer immunotherapy made a significant breakthrough due to the development of adoptive T cell therapies and the use of monoclonal antibodies blocking the CTLA-4 and programmed cell death 1 (PD1) immune checkpoints [1–7]. However, in both cases, harmful autoimmune side effects have been observed, which likely result from the breakage of peripheral tolerance to antigens expressed by normal tissues.

The molecular nature of the antigens recognized by CTL on tumors was revealed in 1989, when Lurquin et al. showed that a mouse tumor-specific CTL recognized a peptide derived from a self-protein, mutated in cancer cells [8]. This observation demonstrated that MHC class I molecules continuously display on the cell surface peptides of 8 to 10 amino acids that are derived from a wide variety of, if not all, intracellular proteins [9]. In tumors, some of these peptides originate from altered or aberrantly expressed proteins, thereby marking the cells for CTL recognition [10].

Here, I review the nature of the peptides that are recognized by antitumor CTL together with the processes involved in their presentation to the immune system. I also discuss their use in the context of cancer immunotherapy.

## 2. Human Tumor Antigens

### 2.1. Identification of Tumor Antigens

Antitumor CTL clones have been isolated from the blood or tumors of cancer patients [11, 12]. One approach often employed to identify the peptides recognized by such CTL is expression cloning, which consists in isolating the peptide-encoding gene by transfecting a library of tumoral cDNA and testing the transfected cells for their ability to activate the CTL clone [13, 14]. Fragments of the identified gene can then be transfected to define the region encoding the antigenic peptide, and finally candidate peptides bearing adequate HLA-binding motifs are tested for their ability to sensitize target cells to lysis by the CTL. This approach was successfully used to identify a large number of antigenic peptides [13, 15–17].

Nowadays, tumor-associated antigenic peptides are often identified using the “reverse immunology” approach [18], which consists in selecting peptides with adequate HLA-binding motifs inside a protein of interest, such as proteins encoded by mutated oncogenes or genes that are either selectively expressed or overexpressed by tumors. Candidate peptides are synthesized and tested for HLA binding* in vitro*. The most efficient binders are pulsed onto antigen-presenting cells, which are used to stimulate T lymphocytes* in vitro*, in order to derive CTL lines or clones that recognize peptide-pulsed target cells. A drawback of this approach is that the identified peptides might not be processed efficiently by tumors. It is therefore essential to verify that the CTL do recognize tumor cells that naturally express the peptide-encoding gene. Additionally, one should test transfectants that express normal levels of the gene [19] or cells where expression of the gene has been knocked down using si or shRNAs [20].

A third approach to antigen identification is based on the elution of antigenic peptides from MHC class I molecules immunopurified from the surface of tumor cells [21–24]. The direct identification by mass spectrometry of the sequence of the eluted peptides is technically demanding but proved useful to identify or to confirm the relevance of peptides that have undergone posttranslational modifications such as serine/threonine phosphorylation [25, 26], glycosylation-dependent asparagine deamidation [27], or peptide splicing [28].

**Figure 1:**
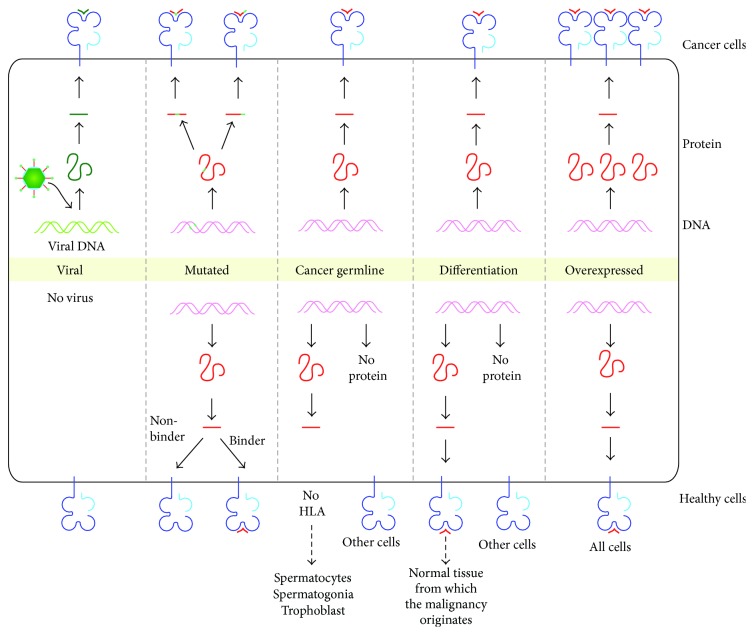
Tumor antigens recognized by cytolytic T lymphocytes. Tumor antigens are classified according to the pattern of expression of the parental gene. The production of antigenic peptides by cancer cells (upper panel) and healthy cells (lower panel) is depicted. Viral antigens are only expressed in virally infected cells. Mutated genes can give rise to a modified peptide that is able to bind the HLA class I molecules while the wild-type peptide cannot (left). The mutation can also alter a peptide, which is able to bind the HLA class I molecule, so that this modified peptide is now recognized as nonself by circulating CTL. Cancer-germline genes are expressed in tumors or germline cells as a result of whole genome demethylation. MAGE-type antigens encoded by cancer-germline genes are not expressed at the surface of healthy cells nor on germline cells since the latter do not express HLA class I molecules. Differentiation antigens are encoded by genes with a tissue-specific expression. They are therefore expressed by some types of tumors and the corresponding healthy tissue. Some genes are overexpressed in tumors as a result of increased transcription or gene amplification. The resulting peptides are highly expressed on these tumors but also show a low level of expression in some or all healthy tissues.

A large number of antigenic peptides recognized by antitumor CTL have been identified using these various approaches. These antigens are conveniently classified according to the expression pattern of the parent gene [10, 29, 30] (Figure 1). A regularly updated database of those antigenic peptides effectively presented by tumor cells can be found on the http://www.cancerimmunity.org/ website [18].

### 2.2. Antigens with High Tumor Specificity

Antigens of three classes can induce tumor-specific T cell responses because they display a tumor-specific pattern of expression [30]: antigens derived from viral proteins, antigens derived from point mutations, and antigens encoded by cancer-germline genes (Figure 1).

#### 2.2.1. Viral Antigens

Viruses are at the origin of several types of cancers including cervical carcinoma, nasopharyngeal carcinoma, hepatocarcinoma, and some leukemias [31]. Viral proteins are produced inside the tumor cells and therefore give rise to antigenic peptides that can be detected by T cells (Figure 1). Vaccines containing long HPV peptides recently emerged as a promising therapeutic modality for HPV-related cancers, as these long peptides proved capable of increasing the number and activity of HPV-16-specific CD4 and CD8 T cells [32, 33].

#### 2.2.2. Antigens Encoded by Mutated Genes

Many CTL isolated from the blood or tumors of cancer patients were found to recognize antigens that arise from point mutations in ubiquitously expressed genes [34–36]. In most cases, the mutation changes one amino acid in the peptide sequence, either enabling the peptide to bind to the MHC class I molecule or creating a new antigenic determinant that is recognized by the CTL (Figure 1). In some cases, the mutation causes a frameshift leading to the production of a new antigenic peptide [37, 38]. Some mutated antigenic peptides result from oncogenic mutations. A mutation in gene* CDK4* was shown to affect the binding of CDK4 to its inhibitor p16/INK4a, thereby favoring uncontrolled cell division [39]. A mutation in gene* CTNNB1* produces an antigenic peptide [40], stabilizes *β*-catenin, and increases its association with the transcription factor Lef-Tcf. This may result in the persistent transactivation of genes involved in melanoma progression [41]. A mutation in gene* CASP8*, recognized by a CTL, was shown to decrease cell sensitivity to apoptosis [42]. In most cases however, these mutations are passenger mutations and the corresponding antigenic peptides are unique to the tumors in which they were identified. Tumors with a high mutation rate, such as melanoma, lung carcinoma, or microsatellite instability (MSI)^+^ colorectal carcinoma are expected to bear more mutated antigens and are therefore more immunogenic. Noteworthy in some patients, the antitumor CTL response is directed mostly against mutated epitopes [43].

Surprisingly, only a few CD8 T cells recognizing peptides derived from genes that are often mutated in cancer, such as* P53*,* KRAS*, or* NRAS*, were isolated [44–46]. Most of these CTL were found using the reverse immunology approach and not the mixed-lymphocyte tumor culture, suggesting that the corresponding peptides might be poorly immunogenic. Peptides derived from chromosomal translocations such as BCR-ABL or ETV6-AML1 were also identified [47, 48].

#### 2.2.3. Cancer-Germline Genes

Another important source of tumor specific antigens is the cancer-germline genes (Figure 1). They include the melanoma-antigen encoding (MAGE) genes, comprising 25 functional genes clustered in three regions of the X chromosome:* MAGEA*,* MAGEB*, and* MAGEC* [49–51]. Other cancer-germline gene families on the X chromosome include the* BAGE* [52],* GAGE* [53, 54],* LAGE*/*NY-ESO1* [55, 56], and* SSX* genes [57, 58]. Cancer-germline genes are expressed in a wide variety of cancer types and not in normal tissues except germline and trophoblastic cells [13, 59]. Their tumor-specific pattern of expression results from the demethylation of their promoter sequence, as part of a genome-wide demethylation that takes place in male germ cells and in some advanced cancers [60–65]. Because male germline cells and trophoblastic cells do not display MHC class I molecules on their surface [66], they cannot display antigens to T cells. The antigenic peptides derived from cancer-germline genes, also called MAGE-type antigens, therefore appear to be strictly tumor-specific and their use as immunotherapeutic targets should not be deleterious to the patient. It is important to note, however, that a low level of expression of MAGE-A12 was recently reported in brain cells [67]. Besides cancer-germline genes, a few examples of aberrant transcripts expressed in tumors but silent or expressed at very low levels in normal tissues have been shown to encode antigenic peptides [68–70]. Recently, peptides were identified that derive from cyclin-A1, a protein with pro-proliferative and anti-apoptotic properties, which is expressed in testis and acute myeloid leukemia [71].

### 2.3. Antigens with Low Tumor Specificity

This category of antigens comprises differentiation antigens and antigens derived from genes that are overexpressed in tumors [30] (Figure 1).

#### 2.3.1. Differentiation Antigens

Differentiation antigens are derived from proteins that are expressed in a given type of tumor and the corresponding healthy tissue. Most identified differentiation antigens are present on melanoma cells, in which the corresponding protein is often involved in melanin biosynthesis or melanosome biogenesis. The expression of the corresponding genes depends on transcription factor MITF (microphthalmia associated transcription factor) [72]. Interestingly, spontaneous responses to peptides derived from proteins such as tyrosinase [15, 73], gp100/pmel17 [19, 74, 75], Melan-A/MART-1 [76, 77], gp75/TRP1 [78], or TRP2 [79] are frequent in melanoma patients and healthy donors [80, 81], suggesting that central tolerance to these antigens is not complete. T cell responses to differentiation antigens can lead to vitiligo, a partial skin depigmentation often observed in melanoma patients and generally associated with a good prognosis [82–84]. Peptides were also identified from the prostate specific antigen and the prostatic acidic phosphatase, two proteins expressed in normal prostate and tumoral prostate tissues [85, 86]. Finally, the carcinoembryonic antigen (CEA) is often highly expressed in colorectal cancer and other epithelial tumors but is also present at lower level in a variety of normal epithelial cells of the intestinal tract [87].

#### 2.3.2. Overexpressed Antigens

Overexpressed antigens also provide candidates for the development of immunotherapeutic vaccines. The difficulty with these antigens is the reliability of the quantification of their amounts on the surface of tumoral versus normal cells, on the basis of which one predicts that there might be a threshold of expression below which the CTL will not recognize the antigen. A number of antigenic peptides have been reported to be “overexpressed,” most of which identified using the reverse immunology approach [18]. An interesting example of overexpressed antigen is the peptide recognized by a CTL on a renal cell carcinoma and encoded by gene* MOK* (*RAGE-1*) [88].* RAGE-1* is expressed in tumors of different histological types but is silent in normal tissues except retina, where low expression is observed. Because the eye is an immunologically privileged site [89, 90] and because retina cells do not seem to express MHC class I molecules [91], immunization against RAGE-1 could be considered against renal cell carcinoma, which express no cancer-germline genes. At least five peptides recognized by CTL in melanoma patients were shown to derive from* PRAME*, a gene overexpressed in a number of tumor types, but expressed at low levels in various normal tissues [92, 93]. Other examples of peptides derived from overexpressed genes include those derived from the inhibitor of apoptosis protein survivin [94, 95], the wild-type p53 protein [96, 97], or the oncogene and growth factor ERBB2 (HER2/NEU) which is overexpressed in many epithelial tumors such as ovarian and breast carcinoma due to increased gene transcription and gene amplification [98–100]. Peptides were also identified that derive from the protein Wilms tumor 1 (WT1), a transcription factor expressed at 10- to 1000-fold higher levels in leukemic versus normal cells [101–103]. A decrease in the number of leukemic cells was observed in leukemia patients following allogeneic stem cell transplantation and injection of donor-derived CTL recognizing the HLA-A2-restricted peptide WT1_126–134_, without evidence of autoimmune toxicity [104]. As overexpressed antigens are shared by numerous tumors, they represent attractive targets for the development of immunotherapy; however their use is not devoid of the risk of developing autoimmune reactions due to the low but still detectable expression of the corresponding genes in healthy tissues.

### 2.4. The Importance of Tumor Specificity in Cancer Immunotherapy

Although antibodies targeting the immune checkpoints CTLA-4 and PD1 proved efficient at inducing sustained clinical responses in cancer patients, harmful autoimmune side effects have been observed in a large number of patients. This damage to healthy tissues likely results from the fact that checkpoints inhibitors boost overall T cell immunity, thereby unleashing peripheral tolerance to antigens expressed by these tissues. The T cells responsible for these toxicities were not yet characterized but they likely correspond to autoreactive T cells that are not specific for the tumor.

To be safe and efficient, immunotherapy strategies should therefore elicit efficient T cell responses against antigenic peptides that are present on tumor cells but not on healthy cells in order to avoid such autoimmune side effects. In that regard, antigens encoded by mutated genes are among the safest. So far, their identification relied mostly on the screening of autologous cDNA libraries [18]. In melanoma patients, about one half of the currently defined tumor-specific antigens recognized by CTL are mutated antigens, while the other half correspond to MAGE-type antigens [30]. Using whole genome sequencing of tumor samples, Robbins et al. have speeded up the identification procedure of antigens derived from passenger mutations recognized by tumor infiltrating lymphocytes (TIL) [105]. Adoptive transfer of these TIL was followed by objective tumor regressions [105, 106]. Because of the therapeutic potential of mutated antigens, identification approaches based on exome sequencing or mass spectrometry might lead to patient-tailored procedures.

For the nonmutated antigens, strict tumor specificity is often hard to certify. Even if an antigen shows a tumor-specific pattern of expression, one can never exclude that it could have a substantial degree of expression in a small subset of cells of the body. No toxicity was observed in cancer vaccine trials based on the activation of patient T cells against such antigens; however, vaccination approaches generally induce only low amounts of CTL [107]. The risk is much higher, for example, with adoptive T cell therapy, which is based on the infusion of a large number of high avidity T cells. About 30% of melanoma patients infused with T cells transduced with a high affinity receptor recognizing melanoma differentiation antigens Melan-A/MART1 or gp100 showed objective cancer regression, while 50% of the patients developed vitiligo and sometimes destruction of melanocytes in the eye and inner ear [108]. The transfer of T cells engineered to express a single-chain murine antibody-type receptor recognizing carbonic anhydrase IX, a protein present on renal cell carcinoma but also on bile epithelial cells, was found to encounter liver toxicity [109]. Recent clinical trial based on the adoptive transfer of T cells engineered to express a murine TCR recognizing the carcinoembryonic antigen led to objective tumor regression in one patient out of four; however, all patients treated developed severe colitis [110]. Finally, transferring T cells carrying a chimeric receptor against ERBB2 was shown to be lethal, due to the rapid gathering of the infused T cells at the lung epithelium, where ERBB2 is expressed at low level [111]. One should therefore ensure that the antigenic peptide targeted by such therapies is strictly specific to the tumor. This is particularly the case when using TCR engineered to increase their affinity, because they bypass the mechanisms responsible for the establishment of natural immune tolerance. Moreover, problems of cross-reaction with unrelated peptides expressed on healthy tissues can also arise. In a recent trial, administration to patients of T cells engineered to express an affinity-enhanced TCR recognizing the peptide EVDPIGHLY derived from MAGE-A3 resulted in a serious adverse event and fatal toxicity against cardiac tissue, probably caused by a cross-recognition of the peptide ESDPIVAQY derived from the muscle protein TITIN [112, 113]. In another trial, adoptive transfer of T cells engineered to recognize the MAGE-A3 peptide KVAELVHFL shared by MAGE-A9 and closely related to epitopes from MAGE-A2, MAGE-A6, and MAGE-A12, three patients experienced mental status changes, and two patients lapsed into comas and subsequently died [67]. This was most likely related to a previously unrecognized expression of MAGE-A12 in the brain, which resulted in the destruction of the neuronal tissue by engineered T cells. In this case, the TCR used to engineer the transferred T cells was obtained from an HLA-A2 transgenic mouse immunized using MAGE-A3 and the TCR was further modified to improve peptide reactivity. Moreover, the toxicity of the transfused T cells appeared to be related to the amount of cells transfused. Altogether, these results confirm the antitumor activity of T cells engineered to express TCR and illustrate the risk of this approach when dealing with antigens that are not truly tumor-specific or with manipulated TCR that have bypassed the mechanisms of natural tolerance.

## 3. Processing of Human Tumor Antigens

### 3.1. The MHC Class I Presentation Pathway

**Figure 2:**
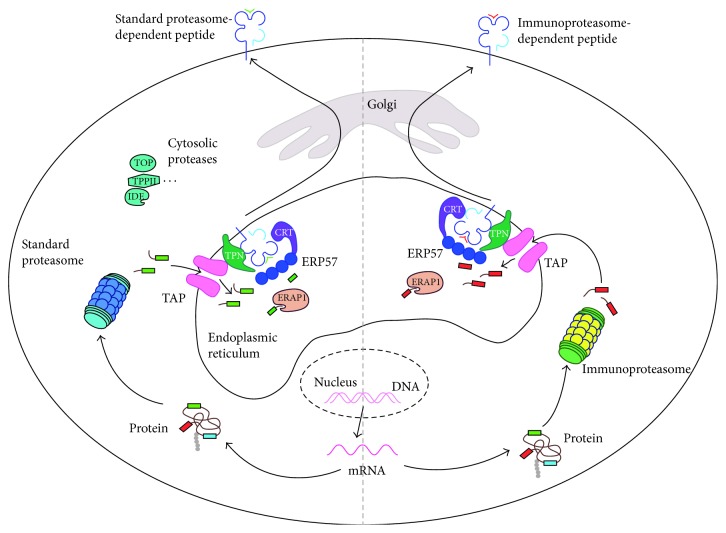
Processing of tumor antigens recognized by CD8^+^ T cells. CTL recognize peptides that are produced by the degradation of cellular proteins by the proteasome. Four types of proteasome exist, two of which are represented here (standard proteasome and immunoproteasome). Peptides resulting from proteasome degradation are then transported in the lumen of the ER by the TAP transporter. Peptides bearing an extended N-terminus can be further trimmed by additional proteases such as ERAP1 before being loaded on HLA class I molecules with the help of the peptide loading complex, which is composed of TAP, tapasin (Tpn), the oxidoreductase ERp57, and the chaperone calreticulin (CRT). Peptide/HLA complexes are then transferred to the cell surface through the secretory pathway.

Antigenic peptides recognized by CTL usually originate from the degradation of cellular proteins by a large cytosolic complex called the proteasome (Figure 2). Peptides released from proteasomal degradation are transferred into the lumen of the endoplasmic reticulum (ER) by the transporter associated with antigen processing (TAP) (Figure 2). Once in the ER, peptides can be further trimmed by aminopeptidases such as ERAP1 and ERAP2. Peptides with suitable size and sequence will bind to MHC class I molecules, with the help of the peptide loading complex (PLC) composed of TAP, tapasin, ERp57, and calreticulin (CRT). Stable peptide/MHC class I complexes will leave the ER and migrate to the cell surface (Figure 2). Most peptide/MHC complexes displayed at the surface of healthy cells are not recognized by T cells as a result of self-tolerance. However, in tumor cells or cells infected by a virus, a new repertoire of peptides is produced, which is derived from viral or tumor-associated proteins and against which an immune response can be mounted. Since CTL can recognize cells bearing as few as 10 peptide/MHC complexes [114], small changes in the cellular protein content can be detected by the immune system.

### 3.2. The Proteasome

**Figure 3:**
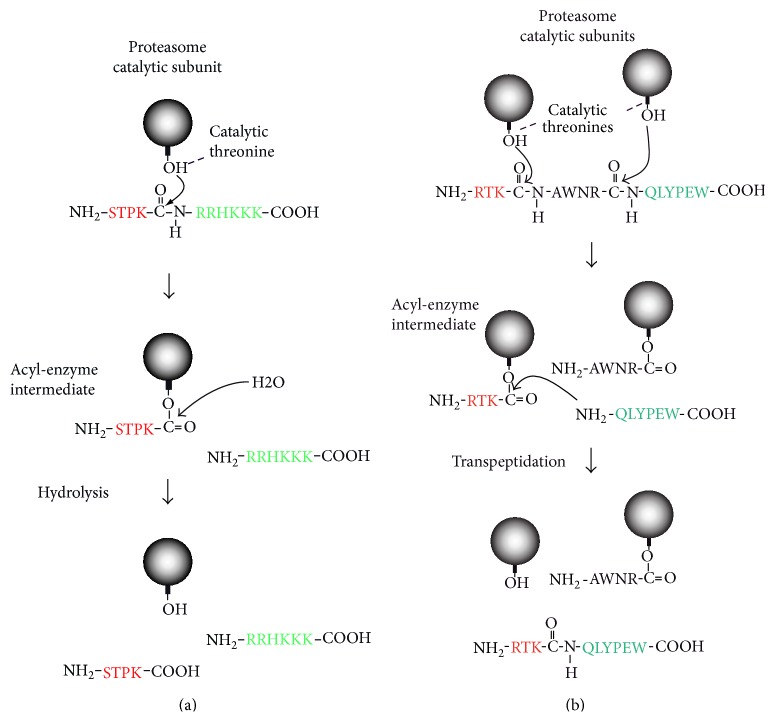
Proteasome activities. (a) Peptide-bond hydrolysis. In the course of peptide-bond hydrolysis, the hydroxyl group of the N-terminal threonine produces a nucleophile attack on the carbonyl of the peptide bond. This leads to the formation of an acyl-enzyme intermediate in which a peptide fragment remains attached to the proteasome through an ester link. Finally hydrolysis of the acyl-enzyme intermediate by a water molecule present in the proteasome chamber will restore the hydroxyl group of the catalytic threonine and release the peptide. (b) Peptide splicing by the proteasome. Splicing of the antigenic peptide RTK_QLYPEW derived from the differentiation antigen gp100. After formation of the acyl-enzyme intermediate involving the fragment RTK, the free N-terminal amino-group of peptide QLYPEW present in the proteasome chamber performs a nucleophilic attack on the acyl-enzyme intermediate. This leads to the creation of a new peptide bond, which assembles both fragments of the spliced peptide. Balls represent the catalytic *β* subunits of the proteasome. The hydroxyl group of the N-terminal threonine is indicated.

The 20S proteasome is a large barrel-shape structure made of four stacked heptameric rings that delimit a catalytic chamber inside which proteins are degraded. The two outer rings of 20S proteasome are made of *α*-subunits (*α*1–7), while the two inner rings contain *β*-type subunits (*β*1–7), three of which (*β*1, *β*2, and *β*5) are catalytic in the vertebrate proteasome. The 20S proteasome associates with the 19S regulatory complex to form the 26S proteasome, a complex responsible for the breakdown of polyubiquitinylated proteins [115]. One of the earliest hints that production of antigenic peptides was dependent on the proteasome was the observation that ubiquitination was necessary for the presentation of the model antigen ovalbumin [116]. Later on, the use of specific cell-permeable proteasome inhibitors, which reversibly or irreversibly bind to the hydroxyl group of the proteasome catalytic subunits, confirmed the proteasome involvement in the production of antigenic peptides and in the overall peptide supply to MHC class I molecules [117–120]. Mutagenesis studies as well as the analysis of crystal structures of inhibitor-bound proteasomes have shown that the hydroxyl group of the N-terminal threonine residue of each catalytic subunit was responsible for the nucleophilic attack that initiates hydrolysis of the peptide bond [121–123]. This nucleophilic attack on the peptide bond leads to the formation of an acyl-enzyme intermediate in which a peptide fragment remains attached to the proteasome by an ester link. This acyl-enzyme is then rapidly hydrolyzed by water molecules found in the catalytic chamber and the released peptide is then transferred back into the cytosol (Figure 3(a)) [124].

Three major types of catalytic activities are associated with proteasome function: the PGPH (peptidyl-glutamyl peptide bond hydrolysing) or caspase-like activity, the trypsin-like activity, and the chymotrypsin-like activity, which cleave after acidic, basic, and hydrophobic residues, respectively. Based on the study of yeast proteasome mutants, the caspase-like activity was linked to the *β*1 subunit, while the trypsin-like and the chymotrypsin-like activities were associated to the *β*2 and *β*5 subunits, respectively [125–130]. However, other parameters are also involved as some proteasome subunits show overlapping specificities [128] and the sequences surrounding the cleavage site also influence cleavage strength [131, 132]. The quality of proteasome degradation will influence the nature of the peptides displayed at the cell surface by MHC class I molecules. A few years ago, my colleagues and I discovered that the proteasome was able to produce antigenic peptides by assembling peptide fragments that are distant in the parental protein. This process, called peptide splicing, takes place inside the catalytic chamber of the proteasome as a consequence of protein degradation (Figure 3(b)). We showed that peptide splicing takes place through a transpeptidation that involves the nucleophilic attack of the acyl-enzyme intermediate by the N-terminus of a peptide fragment present in the chamber [133]. This leads to the creation of a new peptide bond between the C-terminus of the peptide released from the acyl-enzyme intermediate and the N-terminus of the nucleophilic peptide (Figure 3(b)). So far, five spliced antigenic peptides were identified [28, 133–137], three of which were composed of fragments that were assembled in the reverse order to that in which they occur in the parental protein [28, 135, 137]. Studying a spliced peptide derived from gp100 and composed of fragment RSYVPLAH linked to a single arginine (R) at its C-terminus (RSYVPLAH_R), we showed that the attacking nucleophilic peptide required a minimal size of three amino acids in order to perform the splicing reaction. In the case of peptide RSYVPLAH_R, the spliced peptide bears an extended C-terminus that needs to be further trimmed by the proteasome to produce the final antigenic peptide [137]. Mishto et al. recently suggested that, in the catalytic chamber, the nucleophilic peptide would be accommodated in a dedicated pocket, different from the primed substrate binding site. The latter, during proteolysis, accommodates the part of the peptide substrate that is located C-terminally from the cleavage site [138]. The accommodation of the nucleophilic peptide inside a dedicated pocket might facilitate and speed up the peptide splicing process. The existence of this dedicated pocket would explain why, in the study from Mishto et al., the fragments that are the most frequently involved in peptide splicing did not always correspond to the most abundant fragments observed after cleavage of the parental protein, suggesting that affinity of the spliced reactant for this pocket might also rule the peptide splicing reaction [138].

### 3.3. Standard and Immunoproteasome

**Figure 4:**
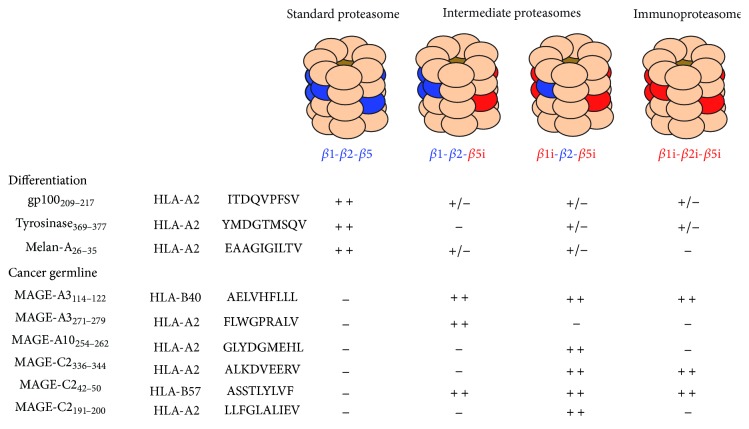
Proteasome subtypes. Mammalian 20S proteasomes are composed four stacked rings of seven subunits each. The two outer rings are made of *α*-subunits and delimit the entrance of the catalytic chamber. The two inner rings are made of *β* subunits, three of which (*β*1, *β*2, and *β*5) are catalytically active. In immune cells or upon induction with IFN*γ*, catalytic subunits *β*1, *β*2, and *β*5 are replaced with their inducible counterparts *β*1i, *β*2i, and *β*5i to form immunoproteasomes. Besides standard and immunoproteasomes, two additional forms of proteasome exist, which contain a mixture of standard and immune catalytic subunits, as indicated. The processing ability of these four types of proteasomes was studied for the indicated peptides (lower part of the figure). ++: efficiently produced, +/−: slightly produced, and −: not produced.

In immune cells and in the presence of inflammatory cytokines, three additional catalytic subunits (LMP-2(*β*1i), MECL-1(*β*2i), and LMP-7(*β*5i)) are expressed, which incorporate into proteasomes instead of subunits *β*1, *β*2, and *β*5, resulting in the formation of immunoproteasomes [139] (Figure 4). The immunoproteasome has an increased propensity to cleave after basic and hydrophobic residues, thereby improving the cell ability to produce peptides for loading on MHC class I molecules, which often require basic and hydrophobic residues at their C-terminus [140–142]. This idea was corroborated by the study of mice knockout for 2 (*β*1i or *β*5i) or 3 immunosubunits, which fail to present a number of MHC class I epitopes [143–145]. Consequently, the peptide repertoire displayed by cells expressing immunoproteasomes is different from that of cells expressing standard proteasomes, and this might have important consequences for the development of cancer immunotherapy strategies [132, 140, 141, 145–147]. Several human tumor antigens were found to be more efficiently produced by the immunoproteasome, such as the HLA-B40-restricted peptide MAGE-A3_114–122_, HLA-A2-restricted peptide MAGE-C2_336–344_, and HLA-B57-restricted peptide MAGE-C2_42–50_ [17, 142, 148–150]. On the other hand, some tumor-associated peptides are better processed by the standard proteasome. The first example, a peptide derived from the ubiquitous protein RU1, was presented to CTL by a renal cell carcinoma but not by the autologous EBV-transformed B cells [118]. This was explained by the fact that, contrary to the tumor cells, EBV-transformed B cells contain a large amount of immunoproteasome, which is not able to produce the antigenic peptide. Since then, several other peptides were found to be processed by the standard proteasome but not the immunoproteasome, among which the HLA-A2-restricted peptides Melan-A/MART-1_26–35_ [118], gp100_209–217_ [149], and tyrosinase_369–377_ [149]. As shown by* in vitro* digestion of long peptide precursors, the lack of processing of antigenic peptides by one proteasome type is generally caused by an internal cleavage that destroys the peptide [142, 149, 150]. Interestingly, studying the differential processing of the spliced peptides by the proteasome, Dalet et al. observed that three spliced peptides were better produced by the standard proteasome, while one peptide was better produced by the immunoproteasome [151]. The efficiency of the peptide splicing reaction depended on the ability of each proteasome type to liberate the peptide fragments involved in the splicing [151].

### 3.4. Intermediate Proteasomes

The existence of intermediate proteasomes, composed of a mixture of standard and immunosubunits, was suggested by the observation that some tissues contain some but not all proteasome immunosubunits [152] (Figure 4). Using a unique set of antibodies directed against proteasome catalytic subunits and recognizing proteasomes in their native configuration, Guillaume et al. demonstrated the existence of intermediate proteasomes containing one (*β*5i) or two (*β*1i *β*5i) immunosubunits [142]. The absence of other types of intermediate proteasomes is in agreement with the rules of proteasome assembly, which depends on the nature of the subunit propeptide [153, 154]. Indeed, the fact that the incorporation of *β*2i depends on the previous incorporation of *β*1i explains why Guillaume et al. did not detect intermediate proteasomes containing *β*2i only [142]. Moreover, the presence of the immunosubunit *β*5i is required for the maturation of proteasomes containing *β*1i and *β*2i [139], explaining why intermediate proteasomes lacking *β*5i were never detected. Interestingly, intermediate proteasomes *β*1-*β*2-*β*5i and *β*1i-*β*2-*β*5i represent 10 to 20% of the total proteasomes found in tumors and 30 to 50% of those found in liver, kidney, small bowel, colon, and dendritic cells [142]. Intermediate proteasomes *β*1-*β*2-*β*5i and *β*1i-*β*2-*β*5i were shown to display chymotrypsin-like and trypsin-like activities that are intermediate between standard and immunoproteasomes [142]. The *β*1 *β*2 *β*5i intermediate proteasome displays a caspase-like (cleavage after acid amino acids) activity similar to that of the standard proteasome, while this activity is low in *β*1i-*β*2-*β*5i proteasome. This originates from the fact that the caspase-like activity is generally assigned to the *β*1 subunit, which is present in standard and intermediate proteasome *β*1-*β*2-*β*5i and absent in immunoproteasome and intermediate proteasome *β*1i-*β*2-*β*5i. Because of their particular cleavage properties, intermediate proteasomes were shown to produce a unique repertoire of peptides. Indeed, some antigenic peptides such as the HLA-A2-restricted peptides MAGE-A10_254–262_ and MAGE-C2_191–200_ are exclusively produced by the intermediate proteasome *β*1i-*β*2-*β*5i while the HLA-A2-restricted peptide MAGE-A3_271–279_ is only produced by intermediate proteasome *β*1-*β*2-*β*5i [142, 150]. Trying to induce T cell responses against antigenic peptides that are produced by both immunoproteasome and intermediate proteasomes should therefore enable the recognition by the immune system of tumors in any circumstances.

### 3.5. Other Proteases Producing Antigenic Peptides

Although most antigenic peptides appear to be produced by the proteasome, others are processed independently from the proteasome through the action of cytosolic proteases such as TPPII or insulin-degrading enzyme [120, 155, 156]. Recently, the production of a PRAME peptide was shown to involve the proteasome for cleavage at the N-terminus while the C-terminus required sequential cleavages by nardilysin and thimet oligopeptidase [157]. Angiotensin-converting enzyme (ACE) was also suggested to play a role in the edition of the carboxyl-terminus of proteasome produced MHC class I peptides [158]. Antigenic peptides produced through proteasome-independent pathways might represent interesting targets in the context of immunotherapy, as some of those antigens might keep being expressed by tumors without being influenced by the surrounding environment.

### 3.6. Central Tolerance to Human Tumor Antigens

Central tolerance to self-antigens is established in the thymus during T cell development, through the sequential steps of positive selection, which occurs in the thymic cortex and retains only T cells whose T cell receptor (TCR) can interact with self-MHC, and negative selection, which eliminates T cells that recognize self-peptides presented in the thymic medulla. Thus, the antitumor T cell repertoire present in the periphery is shaped by the ability of the thymic medulla to process and present tumor-associated antigenic peptides. T cells directed against mutated (and viral) antigens, which are not expressed by the thymic medulla, are not eliminated in the thymus and high affinity T cells to these antigens are therefore present in the periphery, probably explaining the prevalence of the spontaneous antitumor responses to mutated antigens [30, 43]. The presence in the blood of cancer patients of CTL directed against antigens encoded by cancer-germline genes or differentiation antigens indicates that natural immune tolerance against these antigens is either incomplete or absent. Interestingly, antigenic peptides encoded by* MAGE* genes are usually better produced by the immuno- or the intermediate proteasomes than by the standard proteasome (Figure 4) [10, 159]. This contrasts with differentiation antigens, usually produced by the standard proteasome but not by the immuno- or the intermediate proteasomes (Figure 4). The exact proteasome content of thymic epithelial cells is not yet known; however, it was suggested to contain mostly immunoproteasome [160]. For differentiation antigens, the absence in the blood or tumor of cancer patients of T cells recognizing immunoproteasome-dependent peptides probably reflects the efficient central tolerance toward these antigens: T cells directed against immunoproteasome-dependent differentiation peptides do not survive thymic selection. The situation is more complex for antigens encoded by cancer-germline genes. As stated above, these antigens are usually processed by the immunoproteasome or the intermediate proteasomes, suggesting a lack of central tolerance to these antigens. It was initially considered that this lack of central tolerance resulted from the lack of expression of these genes in the thymus. However, Gotter et al. showed that a number of cancer-germline genes were expressed at low levels in medullary thymic epithelial cells (mTEC), which are the cells that present antigens for negative selection [161]. It was then considered that the low expression level of these genes in mTEC might result in partial tolerance, leading to the negative selection of high avidity T cells only. The antitumor T lymphocytes present in the blood of cancer patients would then derive from low avidity T cells that have escaped negative selection in the thymus. To confirm this concept, Huijbers et al. produced knockout mice for the murine cancer-germline gene* P1A* [162]. The expectation was that these mice should contain an unselected anti-P1A T cell repertoire containing high affinity T cells. Surprisingly, while the number of anti-P1A T cells was slightly higher in the P1A-KO mice, their repertoire was barely different from that of wild-type mice, indicating that there is only minimal tolerance against this antigen in WT mice, despite the detectable expression of P1A in mTEC [162]. The apparent lack of central tolerance to the P1A antigen could result from the inability of mTEC to process the P1A peptide. Identifying exactly the type of proteasome present in thymic epithelial cells should therefore shed a better light on the lack of central tolerance to cancer-germline genes and help identifying those antigens for which central tolerance is limited and against which T cells with a relatively high affinity should be found in the periphery.

## 4. Future Challenges for Cancer Immunotherapy

### 4.1. The Potential of Cancer Vaccines

Current developments based on the use of T cell stimulating antibodies or on the adoptive transfer of antitumor T cells have highlighted the power of cancer immunotherapy strategies [1–5, 163]. A significant proportion of the patients treated using these therapies shows remarkable clinical responses and some of these patients even appear disease-free several years after initiation of the treatment. Nevertheless, as discussed above, both therapies often lead to the development of strong autoimmune side effects that need to be controlled. Additionally, although the response to adoptive transfer and stimulating antibodies therapies shows great promises, a large number of patients still remain refractory to these treatments. Therapeutic cancer vaccines aim at specifically activating antitumor CTL already present in the blood or the tumor of cancer patients. They appear safer and better controlled, first because vaccination focuses specifically on the activation of anti-vaccine T cells and second because the T cells targeted by the vaccine have undergone thymic selection, a process that should minimize the occurrence of undesirable immune responses against self-antigens expressed by normal tissues. More specifically, antigenic peptides identified from mixed-tumor lymphocytes cultures, that is, by the* in vitro* stimulation with autologous tumor cells of T cells originating from cancer patients, should be the safest, as they generally target antigens against which a previous spontaneous response has been mounted, usually without any noticeable side effects [30]. On the other hand, the safety of antigens identified using the reverse immunology approach, that is, the* in vitro* priming of healthy donor or cancer patient T cells with pulsed dendritic cells, should be appropriately evaluated, as it is not always known whether these antigens can be safely targeted by the immune system.

No adverse events were observed in the cohorts of patients who were previously vaccinated using peptide, full-length protein or virus containing MAGE-1 or MAGE-3, even in the few responding patients [164–166]. Interestingly, following vaccination, patients who responded to the vaccine were shown to display a considerable enrichment of antitumor T cells in their metastases, when compared to anti-vaccine T cells, suggesting that activation of only a few anti-vaccine CTL can lead to the priming or reactivation of T cells recognizing tumor antigens unrelated to the vaccine [167, 168].

### 4.2. Challenges of Immunotherapy: The Tumor Escape and Immunosuppression

It seems now evident that most melanoma patients develop a spontaneous T cell response to their tumor [12, 168]. Although the presence of TIL at the tumor site is often associated with a good prognosis [169], in many cases, these TIL are overwhelmed by tumor development and they remain inactive and anergized at the tumor site probably explaining why, so far, vaccination of cancer-bearing patients only brought about 5 to 10% clinical responses [29]. The inability of antitumor lymphocytes to eliminate the tumor can be caused by a multiplicity of factors. One of these factors is the loss of those antigens that were initially recognized by T lymphocytes on tumors. This loss of antigen can result from genetic defects affecting either the antigenic peptide, the HLA molecule, or any of the proteins involved in the MHC class I processing machinery. A drastic example is the downregulation of the TAP transporter or tapasin, which is observed in a number of cancer types and affects the processing and presentation of a large number of antigenic peptides [170–174]. Cytokines such as IFN*γ*, which is produced by tumor-infiltrating lymphocytes or NK cells, can also modify the peptide repertoire expressed by tumors. Indeed, although IFN*γ* increases the presentation of a number of antigenic peptides by upregulating the expression of HLA class I heavy chains, *β*2m, TAP1, TAP2, or Tapasin, it also decreases the presentation of those peptides that are destroyed or poorly produced by the standard proteasome [142, 149, 150]. This is true, for example, for peptides derived from melanoma differentiation antigens, which are processed by the standard proteasome but not by the immunoproteasome. Consequently, the following sequence of events might occur: during the initial phase of tumor infiltration by T cells, antitumor lymphocytes attack the tumor cells. The antigens recognized by these lymphocytes are probably mostly produced by standard or intermediate proteasomes, which are expressed by tumors at steady state. These T cells produce INF*γ*, which modifies the proteasome content of the surrounding tumor cells. As a result, tumor cells no longer express the standard-proteasome dependent antigens against which the immune response was initially mounted, and they start expressing immunoproteasome-dependent antigens potentially recognized by other antitumor T cells. During this whole process, under the selective pressure of antitumor CTL, the tumor can sometimes escape the immune response by losing the expression of genes that are necessary for the presentation of peptides to antitumor T cells. Additionally, the IFN*γ* produced by activated lymphocytes at the tumor site induces the expression of immunomodulatory molecules that dampen the immune response and inhibit the function of antitumor CTL. One of these immunomodulatory molecules is the enzyme indoleamine 2,3-dioxygenase (IDO), which creates an immunosuppressive environment by depleting tryptophan and releasing metabolites that inhibit T cell proliferation and function while promoting tumor survival and motility [175]. Another example is the programmed death ligand-1 (PD-L1), whose expression on tumor cells is also stimulated by IFN*γ* and whose engagement with its T cell receptor PD-1 leads to a decrease in T cell proliferation, cytokine production, and T cell adhesion [176]. In that context, reverting local immunosuppression by applying anti-PD1 or anti-PDL-1 neutralizing antibodies concomitantly to cancer vaccines might help maintaining the antitumor T cell responses effective. In preclinical models of melanoma and colon carcinoma, PD-1 blockade enhanced the effectiveness of granulocyte macrophage colony stimulating factor (GM-CSF)-secreting tumor cells immunotherapy by increasing survival of tumor-bearing mice and by enhancing antigen-specific immune responses [177]. In another model of murine melanoma, PD1 blockade combined to 4-1BB stimulation greatly improved vaccination with a recombinant human adenovirus expressing the dopachrome tautomerase antigen and resulted in a complete tumor regression [178]. Finally, another study showed that combination of both CTLA-4 and PD1 blocking antibodies with GM-CSF-expressing tumor cell vaccine also efficiently promoted tumor rejection in mouse models of colon carcinoma and ovarian cancer [179, 180]. This was associated with an increased proliferation, function, and tumor infiltration of tumor-specific CTL. On the other hand, suppressive Tregs were inhibited and found in lower number at the tumor site. These results highlight the potential of combination therapies to enhance the effectiveness of cancer vaccines.

### 4.3. Perspectives for Tumor Vaccines

Trying to overcome issues related to tumor escape and immunosuppression is the current challenge for the development of more efficient immunotherapeutic vaccines. To limit the possibilities of developing antigen-loss variants, cancer vaccines should be designed to activate T cells against a great variety of antigens. One should also favor vaccines targeting antigens that have a high tumor specificity and can be presented by tumors in any circumstance. In that regard, tumor antigens processed by both intermediate proteasomes and immunoproteasomes appear as the best choice, since intermediate proteasomes are found in most tumors at steady state while the immunoproteasome is prominent in tumors exposed to an inflammatory environment. Antigens that can still be expressed when components of the antigen processing machinery (such as TAP, tapasin, or ERAP) are affected should also be considered as relevant targets. TAP-independent antigenic peptides mostly include peptides derived from the signal sequences of ER-targeted proteins and can be released in the ER following cleavage by signal peptidase and signal peptide peptidase [181, 182]. Antigens that are specifically expressed in cells lacking components of the MHC class I processing machinery belong to a special category of antigens called T cell epitopes associated with impaired peptide processing (TEIPP) [183]. The absence of TEIPP antigens at the surface of TAP-positive cells results from the fact that these peptides are underrepresented in the ER lumen, when compared to peptides that are pumped in by TAP [184]. Interestingly and contrarily to what is observed in TAP-knockout mice, thymic epithelial cells from wild-type mice could not process the TEIPP peptides despite efficient expression of the parent protein in thymic cells [184]. As a consequence, negative selection of T cells recognizing such TEIPP antigens should not be achieved in wild-type animals, and TEIPP T cells are expected to display a higher avidity when compared to other antitumor T cells, which underwent negative selection [185]. Identification of novel tumor antigens presented in the absence of TAP or other components of the MHC class I machinery, therefore, represents a new avenue of exploration to overcome tumor escape strategies.

Additionally, the success of cancer vaccines will largely depend on their mode of delivery. Optimally, vaccines should elicit strong CTL responses. In that regard peptide-based or protein-based vaccines are not optimal. On the contrary, viral vector-based vaccines designed to express antigens of interest are very immunogenic as they were shown to activate both the innate and adaptive immune system. More specifically, viral based vaccines efficiently infect DC, which are required for the priming of antitumor CTL. Recombinant viral vectors were found to induce very strong CTL responses associated with antitumoral effects [186]. However, they usually require heterologous prime-boost protocols, where a different vector is used for the priming and the boost, to circumvent the negative effect of vector-specific neutralizing antibodies induced by some vectors after a single injection [186–190].

Finally, as discussed above, reverting local immunosuppression will be a key parameter for the progression of cancer immunotherapy. Indeed, patients responding to the antitumor vaccine might be those whose tumor displays the lowest degree of immunosuppression. The development of efficient inhibitors of immunomodulatory enzymes such as IDO, tryptophan 2,3-dioxygenase (TDO), or arginase has become a major topic of research. Other immunosuppressive factors that could also be targeted are numerous. They include soluble factors such as transforming growth factor-*β* (TGF-*β*), interleukin 10, and galectins or suppressive cells found at the tumor site such as regulatory T cells, tolerogenic dendritic cells, or myeloid-derived suppressor cells. The concomitant delivery of new drugs blocking these immunosuppressive factors together with cancer vaccines, checkpoint blockade inhibitors, or adoptive T cell transfer might show great promises for future therapies.
